# Update: Interim Guidance for the Diagnosis, Evaluation, and Management of Infants with Possible Congenital Zika Virus Infection — United States, October 2017

**DOI:** 10.15585/mmwr.mm6641a1

**Published:** 2017-10-20

**Authors:** Tolulope Adebanjo, Shana Godfred-Cato, Laura Viens, Marc Fischer, J. Erin Staples, Wendi Kuhnert-Tallman, Henry Walke, Titilope Oduyebo, Kara Polen, Georgina Peacock, Dana Meaney-Delman, Margaret A. Honein, Sonja A. Rasmussen, Cynthia A. Moore, Contributors, Contributors, E. Oscar Alleyne, Martina Badell, James F. Bale, Wanda D. Barfield, Richard Beigi, Audina M. Berrocal, Carina Blackmore, Eric C. Blank, Jennifer Bolden Pitre, Coleen Boyle, Erin Conners, Christine Curry, Richard N. Danila, Alberto De La Vega, Roberta L. DeBiasi, Gail J. Demmler-Harrison, Siobhan M. Dolan, Rita W. Driggers, Eric Dziuban, John Eichwald, Catherine Eppes, Nicole Fehrenbach, Meg Fisher, Kimberly B. Fortner, Elizabeth Garbarczyk, Francisco García, Stephanie Gaw, Valerie Godoshian, Ivan A. Gonzalez, Caitlin Green, Dixie D. Griffin, Manda Hall, Amy Houtrow, Mark Hudak, Lisa L. Hunter, David Kimberlin, Linda M. Lawrence, Ellen H. Lee, Rebecca Leeb, Deborah Levine, Claritsa Malave, Yvonne (Bonnie) Maldonado, Lynne Mofenson, Sarah B. Mulkey, Flor M. Munoz, Scott Needle, Chloe Oram, Cassandra G. Pasley, Maria Paz Carlos, Alyssa Pensirikul, Emily E. Petersen, Lawrence Platt, S. Grace Prakalapakorn, Sarah Reagan-Steiner, Jeannie Rodriguez, Elizabeth Rosenblum, Pablo J. Sánchez, Magdalena Sanz Cortes, David J. Schonfeld, Carrie K. Shapiro-Mendoza, Dean E. Sidelinger, V. Fan Tait, Miguel Valencia-Prado, Lisa F. Waddell, Michael D. Warren, Susan Wiley, Eileen Yamada, Marshalyn Yeargin-Allsopp, Fernando Ysern, Christopher M. Zahn

**Affiliations:** ^1^Epidemic Intelligence Service, CDC; ^2^Division of Bacterial Diseases, National Center for Immunization and Respiratory Diseases, CDC; ^3^Eagle Medical Services, LLC; ^4^Chickasaw Nation Industries, Inc; ^5^Division of Vector-Borne Diseases, National Center for Emerging and Zoonotic Infectious Diseases, CDC; ^6^Office of the Director, National Center for Emerging and Zoonotic Infectious Diseases, CDC; ^7^Division of High-Consequence Pathogens and Pathology, National Center for Emerging and Zoonotic Infectious Diseases, CDC; ^8^Division of Reproductive Health, National Center for Chronic Disease Prevention and Health Promotion; ^9^Division of Congenital and Developmental Disorders, National Center on Birth Defects and Developmental Disabilities, CDC; ^10^Division of Human Development and Disability, National Center on Birth Defects and Developmental Disabilities, CDC; ^11^Division of Public Health Information Dissemination, Center for Surveillance, Epidemiology and Laboratory Services, CDC.; National Association of County and City Health Officials; Emory University; University of Utah School of Medicine; CDC; Magee-Women’s Hospital of the University of Pittsburgh Medical Center; Bascom Palmer Eye Institute, University of Miami Miller School of Medicine; Florida Department of Health; Association of Public Health Laboratories; Family Voices, Inc; CDC; New York City Department of Health and Mental Hygiene; University of Miami Miller School of Medicine; Minnesota Department of Health, Council of State and Territorial Epidemiologists; University of Puerto Rico School of Medicine; The George Washington University School of Medicine and Health Sciences; Baylor College of Medicine; Albert Einstein College of Medicine; Johns Hopkins University School of Medicine; CDC; CDC; Baylor College of Medicine; CDC; Unterberg Children’s Hospital at Monmouth Medical Center; University of Tennessee Medical Center; Centers for Medicare & Medicaid Services; Pima County Department of Health; University of California, San Francisco School of Medicine; CDC; University of Miami Miller School of Medicine; CDC; Affinity Pediatrics, Tift Regional Health System; Texas Department of State Health Services, Association of Maternal and Child Health Programs; University of Pittsburgh School of Medicine; University of Florida College of Medicine-Jacksonville; Cincinnati Children’s Hospital; University of Alabama at Birmingham; American Association for Pediatric Ophthalmology and Strabismus; New York City Department of Health and Mental Hygiene; CDC; Harvard Medical School; Health Resources and Services Administration, Puerto Rico Office; Stanford University School of Medicine; Elizabeth Glaser Pediatric AIDS Foundation; The George Washington University School of Medicine and Health Sciences; Baylor College of Medicine; Healthcare Network of Southwest Florida; CDC; Florida Department of Health; Maternal and Child Health Bureau, Health Resources and Services Administration; University of Miami Miller School of Medicine; CDC; David Geffen School of Medicine at University of California, Los Angeles; CDC; Duke University School of Medicine; CDC; National Association of Pediatric Nurse Practitioners, Emory University; American Academy of Family Physicians; University of California San Diego; Nationwide Children’s Hospital; Baylor College of Medicine; University of Southern California; CDC; County of San Diego Health and Human Services Agency; American Academy of Pediatrics; Department of Health of Puerto Rico; March of Dimes; Association of Maternal and Child Health Programs; Tennessee Department of Health; Cincinnati Children’s Hospital Medical Center; California Department of Public Health; CDC; Puerto Rico Chapter, American Academy of Pediatrics; American College of Obstetricians and Gynecologists.

CDC has updated its interim guidance for U.S. health care providers caring for infants with possible congenital Zika virus infection (*1*) in response to recently published updated guidance for health care providers caring for pregnant women with possible Zika virus exposure (*2*), unknown sensitivity and specificity of currently available diagnostic tests for congenital Zika virus infection, and recognition of additional clinical findings associated with congenital Zika virus infection. All infants born to mothers with possible Zika virus exposure* during pregnancy should receive a standard evaluation at birth and at each subsequent well-child visit including a comprehensive physical examination, age-appropriate vision screening and developmental monitoring and screening using validated tools (*3*–*5*), and newborn hearing screen at birth, preferably using auditory brainstem response (ABR) methodology (*6*). Specific guidance for laboratory testing and clinical evaluation are provided for three clinical scenarios in the setting of possible maternal Zika virus exposure: 1) infants with clinical findings consistent with congenital Zika syndrome regardless of maternal testing results, 2) infants without clinical findings consistent with congenital Zika syndrome who were born to mothers with laboratory evidence of possible Zika virus infection,^†^ and 3) infants without clinical findings consistent with congenital Zika syndrome who were born to mothers without laboratory evidence of possible Zika virus infection. Infants in the first two scenarios should receive further testing and evaluation for Zika virus, whereas for the third group, further testing and clinical evaluation for Zika virus are not recommended. Health care providers should remain alert for abnormal findings (e.g., postnatal-onset microcephaly and eye abnormalities without microcephaly) in infants with possible congenital Zika virus exposure without apparent abnormalities at birth.

## Congenital Zika Virus Infection

Zika virus infection during pregnancy can cause serious fetal brain anomalies and microcephaly (*7*). Among infants with substantial loss of brain volume, severe microcephaly and partial collapse of the bones of the upper skull or cranium produce a distinctive physical appearance. Characteristic findings in the brain and spinal cord include thin cerebral cortices with enlarged ventricles and increased extra-axial fluid collections, intracranial calcifications particularly between the cortex and subcortex, abnormal gyral patterns, absent or hypoplastic corpus callosum, hypoplasia of the cerebellum or cerebellar vermis, and hypoplasia of the ventral cord (*8*–*10*). Reported anomalies of the anterior and posterior eye include microphthalmia, coloboma, intraocular calcifications, optic nerve hypoplasia and atrophy, and macular scarring with focal pigmentary retinal mottling (*11*–*13*). Some infants with suspected congenital Zika virus infection without structural eye lesions have cortical visual impairment, attributable to abnormalities in the visual system of the brain (*13*). Other reported neurologic sequelae include congenital limb contractures, dysphagia, sensorineural hearing loss, epilepsy, and abnormalities of tone or movement, including marked hypertonia and signs of extrapyramidal involvement (*14*,*15*). Currently, there is no evidence suggesting that delayed-onset hearing loss occurs following congenital Zika virus infection. Since publication of the previous interim guidance in August 2016 (*1*), additional clinical findings have been reported in the setting of laboratory evidence of Zika virus infection in the mother or infant, including eye findings in infants without microcephaly or other brain anomalies (*16*), postnatal-onset microcephaly in infants born with normal head circumferences (*17*), postnatal-onset hydrocephalus in infants born with microcephaly (*18*), abnormalities on sleep electroencephalogram (EEG) in some infants with microcephaly who did not have recognized seizures (*19*), and diaphragmatic paralysis in infants born with microcephaly and arthrogryposis (*20*–*22*).

## Zika Virus Laboratory Testing

Laboratory testing for Zika virus has a number of limitations. Zika virus RNA is only transiently present in body fluids; thus, negative nucleic acid testing (NAT) does not rule out infection. Serologic testing is affected by timing of sample collection: a negative immunoglobulin M (IgM) serologic test result does not rule out infection because the serum specimen might have been collected before the development of IgM antibodies, or after these antibodies have waned. Conversely, IgM antibodies might be detectable for months after the initial infection; for pregnant women, this can make it difficult to determine if infection occurred before or during a current pregnancy. In addition, cross-reactivity of the Zika virus IgM antibody tests with other flaviviruses can result in a false-positive test result, especially in persons previously infected with or vaccinated against a related flavivirus, further complicating interpretation (*23*,*24*). Limitations of Zika virus IgM antibody assays that were approved under an Emergency Use Authorization have been recognized; both false-positive and false-negative test results have occurred. CDC is updating the Emergency Use Authorization to improve assay performance and develop more standardized methods to improve precision (*25*). Recent epidemiologic data indicate a declining prevalence of Zika virus infection in the Americas; lower prevalence results in a lower pretest probability of infection and a higher probability of false-positive test results.

## Updated Guidance for Testing of Pregnant Women with Possible Zika Virus Exposure

Given the decreasing prevalence of Zika virus infection cases in the Americas and emerging data regarding Zika virus laboratory testing, on July 24, 2017, CDC published updated guidance for testing of pregnant women with possible Zika virus exposure (*2*). Zika virus NAT testing should be offered as part of routine obstetric care to asymptomatic pregnant women with ongoing possible Zika virus exposure (residing in or frequently traveling to an area with risk for Zika virus transmission); serologic testing is no longer routinely recommended because of the limitations of IgM tests, specifically the potential persistence of IgM antibodies from an infection before conception and the potential for false-positive results. Zika virus testing is not routinely recommended for asymptomatic pregnant women who have possible recent, but not ongoing, Zika virus exposure; however, guidance might vary among jurisdictions (*2*). The updated guidance for maternal testing (*2*) is intended to reduce the possibility of false-positive results in the setting of the lower pretest probability; however, there is a possibility that the lack of routine testing might delay identification of some infants without clinical findings apparent at birth, but who may have complications from congenital Zika virus infection. Communication regarding possible maternal exposures between pediatric health care providers and obstetric care providers is critical, and strategies to enhance coordination of care and communication of health information are being developed. For families of infants with possible congenital Zika virus infection, health care providers should ensure that psychosocial support is in place and that families have access to care. The long-term prognosis for infants with congenital Zika virus infection is not yet known; health care providers should strive to address families’ concerns, facilitate early identification of abnormal findings, and refer infants for neurodevelopmental follow-up and therapy when indicated.

## Forum on the Diagnosis, Evaluation, and Management of Zika Virus Infection Among Infants

On August 30–31, 2017, CDC, in collaboration with the American Academy of Pediatrics and the American College of Obstetricians and Gynecologists, convened the Forum on the Diagnosis, Evaluation, and Management of Zika Virus Infection among Infants, with the goal of obtaining individual expert opinion to inform development of updated guidance for diagnosing, evaluating, and managing infants with possible congenital Zika virus infection and to identify strategies to enhance communication and coordination of care of mothers and infants affected by Zika virus. Experts from various medical specialties, professional organizations, public health agencies, and federal agencies participated in the Forum (Box 1). Discussion focused on the diagnosis, evaluation, and management of three groups of infants born to mothers with possible Zika virus exposure during pregnancy: 1) infants with clinical findings consistent with congenital Zika syndrome, regardless of maternal testing results, 2) infants without clinical findings consistent with congenital Zika syndrome who were born to mothers with laboratory evidence of possible Zika virus infection, and 3) infants without clinical findings consistent with congenital Zika syndrome who were born to mothers without laboratory evidence of possible Zika virus infection (Figure).

BOX 1 **Areas of expertise and organizations represented at the Forum on the Diagnosis, Evaluation, and Management of Zika Virus Infection among Infants — Atlanta, Georgia, August 30–31, 2017**

**Specialties represented**
  - Audiology
  - Clinical genetics
  - Developmental and behavioral pediatrics
  - Infectious disease
  - Maternal-fetal medicine
  - Neonatology
  - Neurology
  - Obstetrics and gynecology
  - Ophthalmology
  - Pediatrics
  - Pediatric rehabilitation and medicine
  - Radiology
**Professional organizations**
  - American Academy of Pediatrics (including representation from the Puerto Rico chapter)
  - American College of Obstetricians and Gynecologists
  - Association of Maternal and Child Health Programs
  - Association of Public Health Laboratories
  - Association of State and Territorial Health Officials
  - Council of State and Territorial Epidemiologists
  - Family Voices
  - March of Dimes
  - National Association of County and City Health Officials
  - National Association of Pediatric Nurse Practitioners
**Public health organizations**
  - California Department of Public Health
  - County of San Diego Health and Human Services Agency
  - Department of Health of Puerto Rico
  - Florida Department of Health
  - New York City Department of Health and Mental Hygiene
  - Texas Department of State Health Services
**Federal agencies**
  - Administration for Children and Families
  - Centers for Disease Control and Prevention
  - Centers for Medicare & Medicaid Services
  - Maternal and Child Health Bureau, Health Resources and Services Administration
  - National Institute of Child Health and Human Development, National Institutes of Health
  - Office of the Assistant Secretary for Preparedness and Response

**FIGURE:**
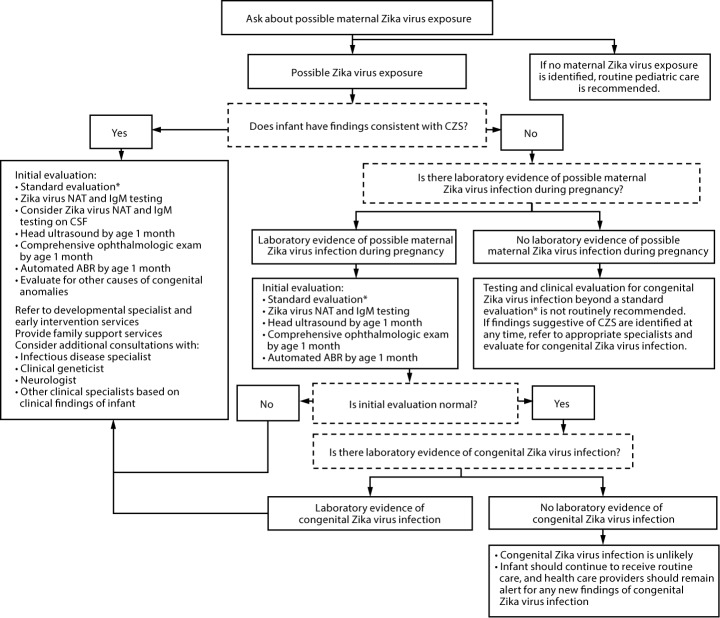
Recommendations for the evaluation of infants with possible congenital Zika virus infection based on infant clinical findings,*^,^^†^ maternal testing results,^§^^,¶^ and infant testing results**^,††^ — United States, October 2017 **Abbreviations:** ABR= auditory brainstem response; CSF = cerebrospinal fluid; CZS = congenital Zika syndrome; IgM = immunoglobulin M; NAT = nucleic acid test; PRNT = plaque reduction neutralization test. * All infants should receive a standard evaluation at birth and at each subsequent well-child visit by their health care providers including 1) comprehensive physical examination, including growth parameters and 2) age-appropriate vision screening and developmental monitoring and screening using validated tools. Infants should receive a standard newborn hearing screen at birth, preferably using auditory brainstem response. ^†^ Automated ABR by age 1 month if newborn hearing screen passed but performed with otoacoustic emission methodology. ^§^ Laboratory evidence of possible Zika virus infection during pregnancy is defined as 1) Zika virus infection detected by a Zika virus RNA NAT on any maternal, placental, or fetal specimen (referred to as NAT-confirmed), or 2) diagnosis of Zika virus infection, timing of infection cannot be determined or unspecified flavivirus infection, timing of infection cannot be determined by serologic tests on a maternal specimen (i.e., positive/equivocal Zika virus IgM and Zika virus PRNT titer ≥10, regardless of dengue virus PRNT value; or negative Zika virus IgM, and positive or equivocal dengue virus IgM, and Zika virus PRNT titer ≥10, regardless of dengue virus PRNT titer). The use of PRNT for confirmation of Zika virus infection, including in pregnant women, is not routinely recommended in Puerto Rico (https://www.cdc.gov/zika/laboratories/lab-guidance.html). ^¶^ This group includes women who were never tested during pregnancy as well as those whose test result was negative because of issues related to timing or sensitivity and specificity of the test. Because the latter issues are not easily discerned, all mothers with possible exposure to Zika virus during pregnancy who do not have laboratory evidence of possible Zika virus infection, including those who tested negative with currently available technology, should be considered in this group. ** Laboratory testing of infants for Zika virus should be performed as early as possible, preferably within the first few days after birth, and includes concurrent Zika virus NAT in infant serum and urine, and Zika virus IgM testing in serum. If CSF is obtained for other purposes, Zika virus NAT and Zika virus IgM testing should be performed on CSF. ^††^ Laboratory evidence of congenital Zika virus infection includes a positive Zika virus NAT or a nonnegative Zika virus IgM with confirmatory neutralizing antibody testing, if PRNT confirmation is performed.

This updated interim guidance is based on current, limited data about Zika virus infection, the interpretation of individual expert opinion collected during the Forum, and knowledge about other congenital infections, and reflects the information available as of September 2017. As more information becomes available, this guidance will be updated.

## Diagnosis of Congenital Zika Virus Infection

The optimal assays, specimens, and timing of testing for congenital Zika virus infection are unknown. A few reports have described infants with clinical findings consistent with possible congenital Zika syndrome but with negative laboratory results (*20*,*26*). Recommended laboratory testing for congenital Zika virus infection includes evaluation for Zika virus RNA in infant serum and urine and Zika virus IgM antibodies in serum. In addition, if cerebrospinal fluid (CSF) is obtained for other purposes, NAT and IgM antibody testing should be performed on CSF because CSF was the only sample that tested positive in some infants with congenital Zika virus syndrome (*26*). Testing of cord blood is not recommended because it can yield false-positive and false-negative test results (*27*,*28*).

Because levels of Zika virus RNA and IgM antibodies decline over time, laboratory testing of infants should be performed as early as possible, preferably within the first few days after birth, although testing specimens within the first few weeks to months after birth might still be useful (*17*,*29*,*30*). Diagnosis of congenital Zika virus infection is confirmed by a positive Zika virus NAT result (Table). If Zika virus IgM antibodies are detected in the infant with a negative NAT, the infant is considered to have probable congenital Zika virus infection. If neither Zika virus RNA nor Zika IgM antibodies is detected on the appropriate specimens (e.g., serum or urine) obtained within the first few days after birth, congenital Zika virus infection is unlikely. Distinguishing between congenital and postnatal infection is difficult in infants who live in areas where there is ongoing transmission of Zika virus and who are not tested soon after birth. If the timing of infection cannot be determined, infants should be evaluated as if they had congenital Zika virus infection.

**TABLE:** Interpretation of results of laboratory testing of infant’s blood, urine, and/or cerebrospinal fluid for evidence of congenital Zika virus infection

Infant test result*		Interpretation
NAT	IgM	
Positive	Any result	Confirmed congenital Zika virus infection^†^
Negative	Nonnegative	Probable congenital Zika virus infection^§,¶^
Negative	Negative	Congenital Zika virus infection unlikely^§,^**

**Abbreviations:** IgM = immunoglobulin M; NAT = nucleic acid test.

*Infant serum, urine, or cerebrospinal fluid.

^†^ Distinguishing between congenital and postnatal infection is difficult in infants who live in areas where there is ongoing transmission of Zika virus and who are not tested soon after birth. If the timing of infection cannot be determined, infants should be evaluated as if they had congenital Zika virus infection.

^§^ Laboratory results should be interpreted in the context of timing of infection during pregnancy, maternal serology results, clinical findings consistent with congenital Zika syndrome, and any confirmatory testing with plaque reduction neutralization testing.

^¶^ If Zika virus plaque reduction neutralization test is negative, this suggests that the infant’s Zika virus IgM test is a false positive.

** Congenital Zika virus infection is unlikely if specimens are collected within the first few days after birth and the clinical evaluation is normal; however, health care providers should remain alert for any new findings of congenital Zika virus infection.

The plaque reduction neutralization test (PRNT), which measures virus-specific neutralizing antibodies, can be used to help identify false-positive results (*24*). In the United States and U.S. territories, if the infant’s initial sample is IgM nonnegative (nonnegative serology terminology varies by assay and might include “positive,” “equivocal,” “presumptive positive,” or “possible positive”) and NAT negative, but PRNT was not performed on the mother’s sample, PRNT for Zika and dengue viruses should be performed on the infant’s initial sample if the test is appropriate given the setting. A negative Zika virus PRNT suggests that the infant’s Zika virus IgM test was a false positive (*23*).

PRNT cannot distinguish between maternal and infant antibodies in specimens collected from infants at or near birth; however, based on what is known about other congenital infections, maternal antibodies are expected to become undetectable by age 18 months and might become undetectable earlier (*31*). For infants whose initial sample is IgM nonnegative and Zika virus neutralizing antibodies are detected on either the infant’s specimen at birth or the mother’s specimen, PRNT at age ≥18 months might help confirm or rule out congenital Zika virus infection. However, PRNT cannot be used to determine timing of infection. If PRNT is positive in an infant at age ≥18 months, congenital Zika virus infection is presumed; however, for infants living in or traveling to areas with risk of Zika virus transmission, postnatal infection cannot be excluded. If PRNT is negative at age ≥18 months, congenital Zika virus infection is unlikely. For infants with clinical findings consistent with congenital Zika syndrome who have maternal laboratory evidence of possible Zika virus infection during pregnancy, PRNT at age ≥18 months could be considered if the infant testing results are negative (i.e., negative Zika virus NAT and IgM on infant serum and urine) or if the infant was not tested at birth.

## Updated Recommendations for Diagnosis, Clinical Evaluation, and Management of Infants with Clinical Findings Consistent with Congenital Zika Syndrome Born to Mothers with Possible Zika Virus Exposure in Pregnancy

**Laboratory testing.** Zika virus testing is recommended for infants with clinical findings consistent with congenital Zika syndrome and possible maternal Zika virus exposure during pregnancy, regardless of maternal testing results (Figure). Testing CSF for Zika virus RNA and Zika virus IgM antibodies should be considered, especially if serum and urine testing are negative and another etiology has not been identified.

**Clinical Evaluation and Management**. In addition to a standard evaluation (Box 2), infants with clinical findings consistent with congenital Zika syndrome should have a head ultrasound and a comprehensive ophthalmologic exam^§^ performed by age 1 month by an ophthalmologist experienced in assessment of and intervention in infants. Infants should be referred for automated ABR by age 1 month if the newborn hearing screen was passed using only otoacoustic emissions methodology (*6*). Because infants with clinical findings consistent with congenital Zika syndrome are at risk for developmental delay and disabilities, referrals to a developmental specialist and early intervention service programs are recommended, and family support services should be provided. In addition, the following consultations should be considered: 1) infectious disease for evaluation of other congenital infections and assistance with Zika virus diagnosis, testing, and counseling; 2) clinical genetics for confirmation of the clinical phenotype and evaluation for other causes of microcephaly or congenital anomalies; and 3) neurology by age 1 month for comprehensive neurologic examination and consideration for other evaluations, such as advanced neuroimaging and EEG. Consultations with other clinical specialists should be based on the infant’s clinical findings (Box 3). Health care providers and families might consider fewer consultations for the evaluation of severely affected infants who are receiving palliative care.

BOX 2 **Standard evaluation recommended at birth and during each well visit for all infants with possible congenital Zika virus exposure during pregnancy — United States, October 2017**

- Comprehensive physical exam, including growth parameters
- Developmental monitoring and screening using validated screening tools recommended by the American Academy of Pediatrics (https://www.aap.org/en-us/advocacy-and-policy/aap-health-initiatives/Screening/Pages/Screening-Tools.aspx)
- Vision screening as recommended by the American Academy of Pediatrics Policy Statement “Visual System Assessment in Infants, Children, and Young Adults by Pediatricians” (http://pediatrics.aappublications.org/content/137/1/e20153596)
- Newborn hearing screen at birth, preferably with automated auditory brainstem response

BOX 3 **Consultations for infants with clinical findings consistent with congenital Zika syndrome — United States, October 2017**

**Consider consultation with the following specialists:**
  - Infectious disease specialist for evaluation for other congenital infections (e.g., toxoplasmosis, syphilis, rubella, cytomegalovirus, or herpes simplex virus) and assistance with Zika virus diagnosis, testing, and counseling
  - Neurologist by age 1 month for comprehensive neurologic examination and consideration for other evaluations such as advanced neuroimaging and EEG
  - Ophthalmologist for comprehensive eye exam by age 1 month
  - Clinical geneticist for confirmation of the clinical phenotype and evaluation for other causes of microcephaly or congenital anomalies
  - Early intervention and developmental specialists
  - Family and supportive services
**Additional possible consultations, based on clinical findings of the infant:**
  - Endocrinologist for evaluation of hypothalamic or pituitary dysfunction and consideration for thyroid testing
  - Lactation specialist, nutritionist, gastroenterologist, or speech or occupational therapist for evaluation for dysphagia and management of feeding issues
  - Orthopedist, physiatrist, or physical therapist for the management of hypertonia, clubfoot or arthrogrypotic-like conditions
  - Pulmonologist or otolaryngologist for concerns about aspiration

The initial clinical evaluation, including subspecialty consultations, can be performed before hospital discharge or as an outpatient, taking into account hospital capabilities and needs of the family. Transfer to a facility with access to pediatric subspecialty care typically is not necessary unless there is an urgent clinical need. Health care providers should maintain vigilance for the appearance of other clinical findings associated with congenital Zika syndrome. Diaphragmatic paralysis should be considered in an infant who develops respiratory distress or failure or who fails to wean from a ventilator. Infant feedings should be monitored closely, and if there are signs of swallowing dysfunction, such as difficulty breathing with feeding, coughing or choking during feeding, or extended feeding times, an assessment for dysphagia should be performed (*32*,*33*). Signs of increasing intracranial pressure (e.g., increasing head circumference, irritability, or vomiting) should prompt neuroimaging to assess for postnatal hydrocephalus.

The follow-up care of infants with findings consistent with congenital Zika syndrome requires a multidisciplinary team and an established medical home to facilitate the coordination of care and ensure that abnormal findings are addressed (*34*). At each subsequent well-child visit, all infants should have a standard evaluation (Box 2) along with routine preventive pediatric care and immunizations (*35*), with decisions about further evaluation guided by clinical findings and made in consultation with the family. Follow-up visits with an ophthalmologist after the initial eye examination should be based on ophthalmology recommendations. As a change from the previous guidance (*1*), a diagnostic ABR is no longer recommended at age 4–6 months for infants who passed the initial hearing screen with automated ABR because of the absence of data suggesting delayed-onset hearing loss in infants with congenital Zika virus infection. Additional follow-up will depend on clinical findings in the infant.

## Updated Recommendations for Diagnosis, Clinical Evaluation, and Management of Infants without Clinical Findings Consistent with Congenital Zika Syndrome Born to Mothers with Laboratory Evidence of Possible Zika Virus Infection During Pregnancy

**Laboratory testing.** Zika virus testing is recommended for infants without clinical findings consistent with congenital Zika syndrome born to mothers with laboratory evidence of possible Zika virus infection during pregnancy (Figure).

**Clinical evaluation and management.** In addition to a standard evaluation (Box 2), infants who do not have clinical findings consistent with congenital Zika syndrome born to mothers with laboratory evidence of possible Zika virus infection during pregnancy should have a head ultrasound and a comprehensive ophthalmologic exam performed by age 1 month to detect subclinical brain and eye findings. Further follow-up visits with an ophthalmologist after the initial examination should be based on ophthalmology recommendations. Infants should also be referred for automated ABR by age 1 month if newborn hearing screen was passed using only otoacoustic emissions methodology.

Health care providers should perform a standard evaluation along with routine preventive pediatric care and immunizations (*35*) at each subsequent well-child visit, and they should be vigilant for signs that might be associated with congenital Zika virus infection. If findings consistent with congenital Zika syndrome (e.g., impaired visual acuity/function, hearing problems, developmental delay, or delay in head growth) are identified at any time, referrals to the appropriate specialists should be made and further evaluation should follow recommendations for infants with clinical findings consistent with congenital Zika syndrome (Figure).

**Infants with laboratory evidence of congenital Zika virus infection.** Laboratory evidence of congenital Zika virus infection includes a positive Zika virus NAT or a nonnegative Zika virus IgM with confirmatory neutralizing antibody testing, if PRNT confirmation is performed. Further clinical evaluation for infants with laboratory evidence of congenital Zika virus infection should follow recommendations for infants with clinical findings even in the absence of clinically apparent abnormalities (Figure). As a change from the previous guidance (*1*), a diagnostic ABR at 4–6 months or behavioral audiology at age 9 months is no longer recommended if the initial hearing screen is passed by automated ABR, because of absence of data suggesting delayed-onset hearing loss in congenital Zika virus infection.

**Infants without laboratory evidence of congenital Zika virus infection**. If adequate laboratory testing is performed (e.g., concurrent testing on infant serum and urine within the first few days after birth), there is no laboratory evidence of congenital Zika virus infection (i.e., negative NAT and IgM on infant samples), and the clinical evaluation is normal, then congenital Zika virus infection is unlikely. Infants should continue to receive routine pediatric care, and health care providers should remain alert for any new findings of congenital Zika virus infection.

## Updated Recommendations for Diagnosis, Clinical Evaluation, and Management of Infants without Clinical Findings Consistent with Congenital Zika Syndrome Born to Mothers with Possible Zika Virus Exposure in Pregnancy but without Laboratory Evidence of Possible Zika Virus Infection During Pregnancy

This heterogeneous group includes mothers who were never tested during pregnancy as well as those whose test result could have been negative because of issues related to timing or sensitivity and specificity of the test. Because the latter issues are not easily discerned, all mothers with possible exposure to Zika virus during pregnancy who do not have laboratory evidence of possible Zika virus infection, including those who tested negative with currently available technology, should be considered in this group.

**Laboratory testing.** Laboratory testing for congenital Zika virus infection is not routinely recommended for infants born to mothers in this category based on the unknown risk for infection; the lower likelihood of congenital Zika virus infection as a result of the declining prevalence of Zika virus infection; and limitations of infant laboratory testing. If abnormal findings are identified, these infants should receive further evaluation, including evaluation and testing for congenital Zika virus infection.

**Clinical evaluation and management**. Infants without clinical findings consistent with congenital Zika syndrome born to mothers without laboratory evidence of possible Zika virus infection during pregnancy should have a standard evaluation (Box 2) performed at birth and at each subsequent well-child visit along with routine preventive pediatric care and immunizations (*35*). Health care providers should be alert to the possibility of congenital infection, especially in infants born to mothers with ongoing possible Zika virus exposure during pregnancy.

Further clinical evaluation for congenital Zika virus infection beyond a standard evaluation and routine pediatric care is not routinely indicated. Health care providers can consider additional evaluation in consultation with families, taking into account the infant’s complete physical examination with emphasis on neurologic findings; risks of screening (e.g., identification of incidental findings); and maternal factors, including the presence and timing of symptoms, and type, location, and length of possible Zika virus exposure. Older infants in whom maternal Zika virus exposure was not assessed at birth and who are evaluated later might also have more clinical data available (e.g., neurologic status, development, visual/hearing impairments, or head circumference trajectory) to guide the evaluation. If findings consistent with congenital Zika syndrome are identified at any time, referrals to the appropriate specialists should be made, and subsequent evaluation should follow recommendations for infants with clinical findings consistent with congenital Zika syndrome (Figure).

## Special Considerations for the Prenatal Diagnosis of Congenital Zika Virus Infection

While much has been learned about congenital Zika syndrome, limitations of laboratory testing exist and the full spectrum of congenital Zika virus infection is not yet known. Similar to other congenital infections, prenatal diagnostic evaluation can inform the clinical evaluation of infants with possible Zika virus exposure. Current CDC guidance regarding prenatal diagnosis is reviewed below (*2*); as more data become available, understanding of the diagnostic role of prenatal ultrasound and amniocentesis in the clinical evaluation of congenital Zika syndrome will improve and guidance will be updated.

**Ultrasound.** Routine screening for fetal abnormalities is a component of prenatal care in the United States. Comprehensive ultrasound examination to evaluate fetal anatomy is recommended for all women at 18–22 weeks’ gestation (*36*). However, for the detection of abnormalities associated with congenital Zika virus infection, the sensitivity, specificity, and positive and negative predictive values of ultrasound are unknown. Prenatal ultrasound findings associated with congenital Zika virus infection include intracranial calcifications at the gray-white matter junction, ventriculomegaly, abnormalities of the corpus callosum, microcephaly, and limb anomalies (*10*,*37*). The reliability of ultrasound detection for each of these abnormalities as isolated findings is unknown (*37*,*38*). Limited data suggest that a constellation of ultrasound abnormalities (e.g., microcephaly, ventriculomegaly, or abnormalities of the corpus callosum) identified prenatally in the context of maternal Zika virus exposure correlates with reported structural abnormalities in infants at birth (*20*,*21*,*39*–*43*).

Questions remain about optimal timing of ultrasound among pregnant women with possible maternal Zika virus exposure. Abnormalities have been detected anywhere from 2 to 29 weeks after symptom onset (*39*,*41*,*43*,*44*); therefore, insufficient data are available to define the optimal timing between exposure and initial sonographic screening. Brain abnormalities associated with congenital Zika syndrome have been identified by ultrasound in the second and third trimesters in published case reports (*20*,*39*,*41*,*43*,*44*). Currently, the negative predictive value of serial normal prenatal ultrasounds is unknown. Serial ultrasound monitoring can detect changes in fetal anatomy, particularly neuroanatomy, and growth patterns (*39*,*41*,*44*). CDC previously recommended serial ultrasounds every 3–4 weeks for women exposed during pregnancy with laboratory evidence of Zika virus infection, based upon existing fetal growth monitoring for other maternal conditions (e.g., hypertension or diabetes) (*2*). However, there are no data specific to congenital Zika virus infection to guide these timing recommendations; clinicians may consider extending the time interval between ultrasounds in accordance with patient preferences and clinical judgment. Women with possible exposure but without laboratory evidence of Zika virus infection during pregnancy should receive ultrasound screening as recommended for routine prenatal care. Future data will be used to inform the optimal timing and frequency of ultrasound in pregnant women with possible Zika virus infection.

**Amniocentesis.** The role of amniocentesis for the detection of congenital Zika virus infection is unknown. Data regarding the positive and negative predictive values and optimal timing for amniocentesis are not available. Reports of the correlation between positive Zika test results in amniotic fluid and clinical phenotype or confirmatory infant laboratory testing are inconsistent (*20*,*42*,*45*,*46*). Zika virus RNA has been detected in amniotic fluid specimens; however, serial amniocenteses have demonstrated that Zika virus RNA might only be present transiently (*45*). Therefore, a negative test result on amniotic fluid cannot rule out congenital Zika virus infection. However, if amniocentesis is indicated as part of the evaluation for abnormal prenatal findings, NAT testing for Zika virus should be considered to assist with the diagnosis of fetal infection.

**Summary of prenatal diagnosis of congenital Zika virus infection.** Given the limitations in the available screening modalities and the absence of effective interventions to prevent and treat congenital Zika virus infection, a shared decision-making model is essential to ensure that pregnant women and their families understand the risks and benefits of screening in the context of the patient’s preferences and values. For example, serial ultrasound examinations might be inconvenient, unpleasant, and expensive, and might prompt unnecessary interventions; amniocentesis carries additional known risks such as fetal loss. These potential harms of prenatal screening for congenital Zika syndrome might outweigh the clinical benefits for some patients; therefore, these decisions should be individualized (*47*).

## Footnotes

* Possible Zika virus exposure includes travel to, or residence in an area with mosquitoborne Zika virus transmission or sex without the use of condoms with a partner who has traveled to or resides in an area with mosquitoborne Zika virus transmission.

† Laboratory evidence of possible Zika virus infection during pregnancy is defined as 1) Zika virus infection detected by a Zika virus RNA nucleic acid test (NAT) on any maternal, placental, or fetal specimen (referred to as NAT-confirmed), or 2) diagnosis of Zika virus infection, timing of infection cannot be determined or unspecified flavivirus infection, timing of infection cannot be determined by serologic tests on a maternal specimen (i.e., positive/equivocal Zika virus immunoglobulin M [IgM] and Zika virus plaque reduction neutralization test [PRNT] titer ≥10, regardless of dengue virus PRNT value; or negative Zika virus IgM, and positive or equivocal dengue virus IgM, and Zika virus PRNT titer ≥10, regardless of dengue virus PRNT titer). The use of PRNT for confirmation of Zika virus infection, including in pregnant women, is not routinely recommended in Puerto Rico (https://www.cdc.gov/zika/laboratories/lab-guidance.html).

§ Assessment of visual acuity (if able, responses to teller or grating tests), pupillary response, external examination, anterior segment examination, intraocular pressure measurement if indicated, and dilated fundus examination. After 3–4 months of age, also assess ocular motility, cycloplegia refraction and accommodation by dynamic retinoscopy. If physical abnormalities are present, recommend photo documentation if resources are available. (https://www.aao.org/preferred-practice-pattern/pediatric-eye-evaluations-ppp--september-2012#sectionII.comprehensiveophthamalicexamination).
